# Use of a Total Variation Minimization Iterative Reconstruction Algorithm to Evaluate Reduced Projections during Digital Breast Tomosynthesis

**DOI:** 10.1155/2018/5239082

**Published:** 2018-06-19

**Authors:** Tsutomu Gomi, Yukio Koibuchi

**Affiliations:** ^1^School of Allied Health Sciences, Kitasato University, Sagamihara, Kanagawa, Japan; ^2^Breast and Endocrine Surgery, National Hospital Organization Takasaki General Medical Center, Takasaki, Gunma, Japan

## Abstract

**Purpose:**

We evaluated the efficacies of the adaptive steepest descent projection onto convex sets (ASD-POCS), simultaneous algebraic reconstruction technique (SART), filtered back projection (FBP), and maximum likelihood expectation maximization (MLEM) total variation minimization iterative algorithms for reducing exposure doses during digital breast tomosynthesis for reduced projections.

**Methods:**

Reconstructions were evaluated using normal (15 projections) and half (i.e., thinned-out normal) projections (seven projections). The algorithms were assessed by determining the full width at half-maximum (FWHM), and the BR3D Phantom was used to evaluate the contrast-to-noise ratio (CNR) for the in-focus plane. A mean similarity measure of structural similarity (MSSIM) was also used to identify the preservation of contrast in clinical cases.

**Results:**

Spatial resolution tended to deteriorate in ASD-POCS algorithm reconstructions involving a reduced number of projections. However, the microcalcification size did not affect the rate of FWHM change. The ASD-POCS algorithm yielded a high CNR independently of the simulated mass lesion size and projection number. The ASD-POCS algorithm yielded a high MSSIM in reconstructions from reduced numbers of projections.

**Conclusions:**

The ASD-POCS algorithm can preserve contrast despite a reduced number of projections and could therefore be used to reduce radiation doses.

## 1. Introduction

Digital tomosynthesis combines the benefits of digital imaging [1, 2] with the tomographic benefits of computed tomography to provide three-dimensional (3D) structural information. This technique can easily be performed in conjunction with radiography to reduce both the radiation doses and associated costs. Digital breast tomosynthesis (DBT) thus provides 3D structural information by reconstructing an entire image volume from a sequence of projection-view mammograms acquired within a small number of projection angles over a limited angular range. As DBT reduces the camouflaging effects of overlapping fibroglandular breast tissue, thereby improving the conspicuity of subtle lesions, its use could potentially improve the rate of early breast cancer detection [2–4]. Several digital mammography-based DBT systems have been developed [5], and this technology is the focus of currently ongoing preliminary clinical studies [2, 6].

In previous studies of DBP, Wu et al. evaluated the conventional reconstruction algorithm (filtered back projection; FBP [7]), statistical iterative reconstruction (IR) algorithms (maximum likelihood expectation maximization; MLEM [3]), and simultaneous IR algorithms (the simultaneous iterative reconstruction technique; SIRT [8]). The results led Wu and colleagues to conclude that the MLEM algorithm provides a good balance of image quality between low- and high-frequency features [3]. Other reports have explored various DBT reconstruction methods [7, 9, 10] or have proposed options for suppressing irrelevant plane information and enhancing DBT image quality [11, 12]. Specifically, DBT reconstruction involves inconsistent images limited by a low signal-to-noise ratio consequent to the superposition of several low-exposure projection images. The concurrent loss of plane-relevant details yields reconstructed images with poor contrast.

Two research objectives have been identified as a consequence of the increasing spread of DBT in clinical practice: estimation of the risk of radiation-induced cancer and characterization of the image qualities of DBT systems to understand the similarities and differences with respect to standard two-dimensional (2D) full-field digital mammography (FFDM). Although both objectives remain under debate [13], Ferreira et al. [14] demonstrated an increase in the risk of induced lung cancer with the DBT scan relative to FFDM, especially if the beam energy has not been optimized in terms of the image quality and absorbed dose [15].

Regarding image quality, a factor called quantum mottle causes spatial incident photon fluctuations and, consequently, radiographic image degradation. As quantum mottle increases at lower levels of exposure, reductions in the doses to patients would be restricted by the degree of quantum mottle even in a perfect detector. Noise also affects the visibility and detectability of subtle microcalcifications (MCs) and masses in reconstructed DBT images. Therefore, a new algorithm that improves image quality via suitable processing would further reduce patient doses and improve detection.

To overcome the above-described limitations, several noise suppression techniques for DBT reconstruction have been proposed [16–19]. Recently, an iterative algorithm based on total variation- (TV-) based compressive sensing was developed for volume image reconstruction from tomographic scans [20–24]. TV is defined as the sum of the first-order derivative magnitudes for all pixels in the image, and TV image has been used as a penalty term in iterative image reconstruction algorithms [24]. TV-minimization is an image domain optimization method associated with compressed sensing theory [22, 24]. Adaptive steepest descent projection onto convex sets (ASD-POCS), a TV-minimization iterative reconstruction (IR) algorithm for image reconstruction, provides a partial solution to the problem of constrained TV-minimization [22]. In TV-minimization IR, the addition of a penalty to the data-fidelity-objective function smooths noise in the image while preserving the internal edges [20–25]. Therefore, TV-minimization IR can preserve contrast while reducing both projection data and radiation doses.

In this study, we evaluated the abilities of four reconstruction algorithms to reduce radiation dose from normal and half projections (i.e., thinned-out normal): a novel TV-minimization IR algorithm (ASD-POCS) and three conventional reconstruction algorithms (FBP, statistical IR-MLEM, and SIRT algorithm algebraic IR-simultaneous algebraic reconstruction technique; SART) [23]). Specifically, we compared the level of contrast preservation when reconstructing a reduced number of projections of both breast phantoms and clinical cases.

## 2. Materials and Methods

### 2.1. Digital Breast Tomosynthesis

This study used a DBT system (Selenia Dimensions; Hologic Inc., Bedford, MA, USA) comprising an X-ray tube with a 0.3-mm focal spot (tube target: W, filtration: 0.7-mm aluminum equivalent) and a digital flat-panel amorphous selenium detector. A total acquisition time of 3.7 s and acquisition angle of 15° were set for DBT procedures. Normal projection images were sampled during a single tomographic pass (15 projections), while half projection images (seven projections) were generated by thinning out normal projection data.

### 2.2. Phantom Specifications

The BR3D Phantom (Model 020; Computerized Imaging Reference Systems, Inc., Norfolk, VA, USA), which comprises multiple heterogeneous slabs, is intended to mimic the composition of glandular and adipose tissues and parenchymal patterns in the human breast. The slabs are composed of epoxy resins with X-ray attenuation properties corresponding to 50% glandular or 50% adipose breast tissue. The target slab was surrounded by nontarget slabs (top, 30 mm and bottom, 10 mm).

### 2.3. Radiation Dose Measurement

The following settings were implemented during each radiation dose setup: a reference radiation dose [automatic exposure control (AEC) = exposure condition at 40-mm thickness and predetermined tube voltage and current] at 28 kVp and 50 mA (15 projections). The average glandular dose (AGD) was calculated using the method proposed by Dance et al. [26] and a Piranha dosimeter (RTI Electronics AB, Mölndal, Sweden) to measure radiation exposure. Measured radiation doses were used to convert the established exposure condition into the AGD; the latter value was 1.51 mGy.

### 2.4. Reconstruction Algorithm

In this study, we used MATLAB (Mathworks; Natick, MA, USA) to perform the FBP, SART, MLEM, and ASD-POCS image reconstruction calculations [27]. The reconstruction data comprised real projection data acquired on a DBT system.

Two-dimensional (2D) image filtering, which multiplies the Fourier transform by a Ramp or SL filter kernel, was used to restore the impulse shape of the reconstructed image. A conventional Ramp or SL filter kernel and the FBP algorithm, which generally produces precise 3D reconstruction images [7], were used to yield FBP images in this study. In contrast to the single-step back projection and FBP algorithms, IR algorithms perform a recursive reconstruction [9]. Specifically, IR iteratively updates the unknown linear attenuation coefficients by minimizing errors between the measured and calculated projection data.

Previous studies have investigated algebraic reconstruction technique (ART) methodologies [8]. An ART rapidly converges by updating the linear attenuation coefficients from a single projection value at each time point. However, the least-squares solution can yield considerable noise if the inverse problem is very poorly posed (e.g., limited angle reconstruction). Several improvements to ART have been proposed to address this issue. For instance, modifications of ART may be compatible with other methods, such as SIRT [8], depending on the projection data volume and the method used to update the given estimation. Notably, SART represents a compromise between ART and SIRT that yields acceptable algorithm stability and convergence in the same process. MLEM methods comprising two steps per iteration (e.g., a forward step for DT acquisition process modeling and backward step for reconstructed object updating) have also been proposed. These methods are applied iteratively, such that the reconstructed volume projections computed from an image formation model will resemble the experimental projections.

**Figure 1:**
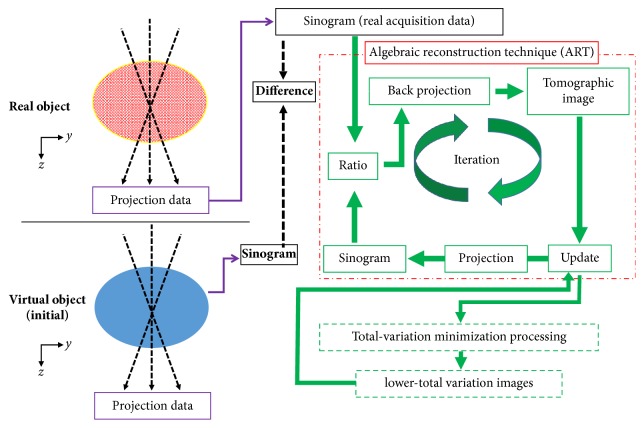
The total variation minimization concept based on the adaptive steepest descent projection onto the convex sets reconstruction algorithm for digital breast tomosynthesis.

**Figure 2:**
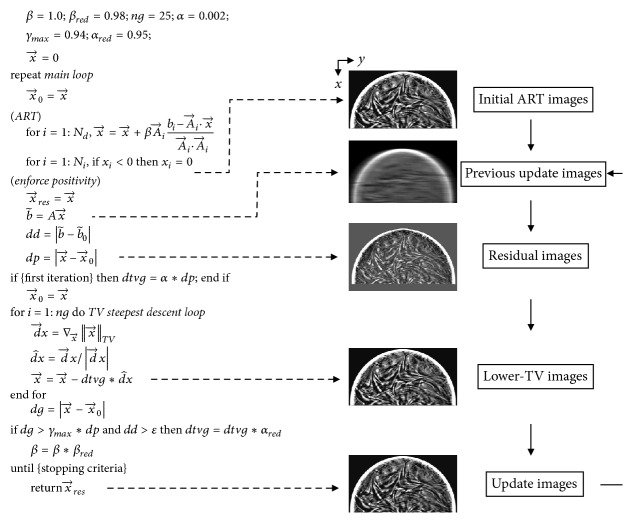
The adaptive steepest descent projection onto the convex sets algorithm in the form of a pseudocode. *β*: the* ART* operator depends on the relaxation parameter. *β*_*red*_: the* ART*-relaxation parameter is reduced by a constant fraction. *α*: total variation (TV) hyperparameter. *α*_*red*_, *γ*_*max*_: these variables control the evolution of *α*. *ng*: iteration number for the TV-steepest descent. Image-space variables are denoted by a vector sign (e.g., $\vec{x}$). Data-space variables are denoted by a tilde (e.g., $\tilde{b}$). The vector ${\vec{A}}_{i}$ is the row of the system matrix that produces the *i*th data element. ${\tilde{b}}_{0}$ is the optimization problem specified by the projection. *ε* is the data-inconsistency-tolerance parameter (reference: Sidky EY, Pan X. Image reconstruction in circular cone-beam computed tomography by constrained, total variation minimization. Phys Med Biol, 2008; 53: p.4788).

Another ART is the ASD-POCS algorithm step that improves data consistency, in which basic projection enforces positivity. ASD-POCS minimizes the TV norm separately in each iteration; in other words, the image is first reconstructed, followed by a reduction in the TV norm at each iteration. To nudge the image toward a minimum-TV solution, POCS steps are alternated with the TV-steepest descent [22]. If the TV-minimization step alone was run during the rest of the algorithms, the result would be a flat image. Alternatively, the ROF model ensures that the image is not significantly altered. The importance of these optimal parameters with respect to image quality has been demonstrated in previous studies [22, 24]. Here, we used optimal parameters for the ASD-POCS algorithms to preserve the edges. Figures 1 and 2 depict the ASD-POCS algorithm in the form of a pseudocode and overview, respectively.

### 2.5. Phantom Evaluation

We calculated the full width at half-maximum (FWHM) and contrast-to-noise ratio (CNR) to evaluate the effects of contrast preservation on each phantom image featured in the in-focus plane. The spatial resolution derived from the FWHM in the in-focus plane (0.29 and 0.40 mm *φ*; CaCO_3_) was evaluated as a quantitative measure of the reconstructed image quality, after which the FWHM of the selected intensity profiles intersecting the three MCs on reconstructed DBT slices were measured. To obtain the intensity profile, three neighboring vertical lines intersecting the MCs (perpendicular to the X-ray sweep direction) were arranged.

**Figure 3:**
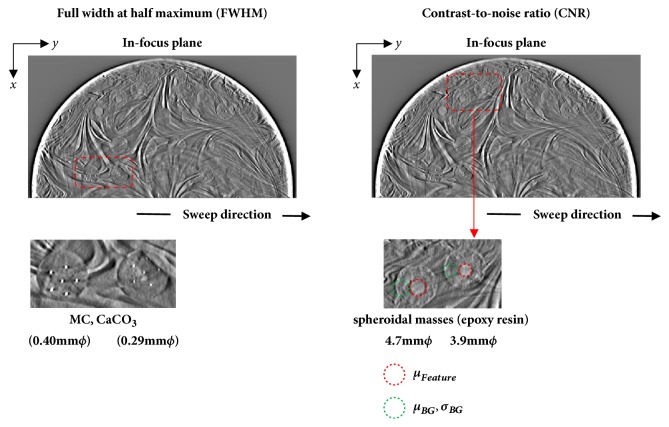
Areas where the full width at half-maximum and contrast-to-noise ratio were measured in a reconstructed image of the BR3D Phantom (in-focus plane).

The contrast derived from the CNR in the in-focus plane [3.9 and 4.7 mm *φ*; spheroidal masses (epoxy resin)] was also evaluated as a quantitative measure of the reconstructed image quality. In tomosynthesis, the CNR is frequently used to estimate low-contrast detectability and was defined in this study as follows:

(1) $$\begin{array}{c} CNR=\frac{\mu_{Feature}-\mu_{BG}}{\sigma_{BG}} \end{array}$$

where *μ*_*Feature*_ is the mean object pixel value, *μ*_*BG*_ is the mean background area pixel value, and *σ*_*BG*_ is the standard deviation of the background pixel values. The latter parameter includes the photon statistics and electronic noise from the results, as well as structural noise that could obscure the object. The sizes of all regions of interest (ROIs) used to measure the CNR were adjusted to an internal signal as shown in Figure 3 (3.9 mm; 21 × 21 pixels, 4.7 mm; 33 × 25 pixels).

### 2.6. Optimization Parameters

**Figure 4:**
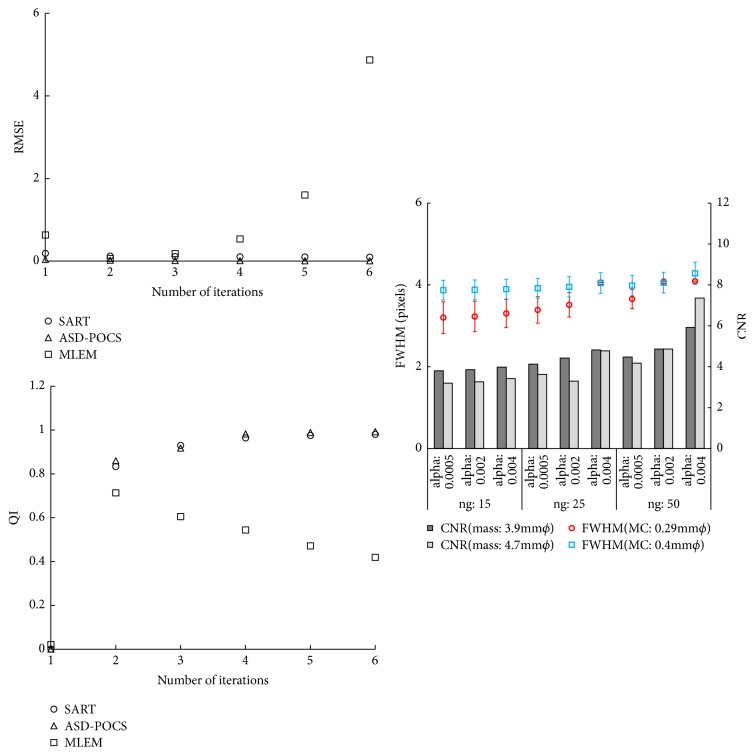
At right, the full width at half-maximum (FWHM) and contrast-to-noise ratio (CNR) characteristics caused by differences in parameters [TV hyperparameter (*α*), iteration number for TV-steepest descent (*ng*)] in the ASD-POCS algorithm (error bar represents the standard error). At left, the root-mean-square error (RMSE) and universal image quality index (QI) characteristics caused by differences in the numbers of iterations in each reconstruction algorithm.

A range of optional parameters have been identified for ASD-POCS [TV hyperparameter (*α*), iteration number for TV-steepest descent (*ng*)]; of these, some are crucial for determining the algorithmic behavior. In this study, we used the FWHM and CNR to verify the optimization of these parameters. To maintain a quality balance between FWHM and CNR performance, a TV hyperparameter (*α*) of 0.002 and iteration number for TV-steepest descent (*ng*) of 25 were selected (Figure 4). We compared the root-mean-square error (RMSE) and universal image quality index (QI) [reconstructed volume image (15 projections) from the previous iteration between the current iteration] to optimize the iteration numbers (*i*) [28]. The QI is mathematically defined by modeling the image distortion relative to the reference image as a combination of three factors: loss of correlation, luminance distortion, and contrast distortion. Because the QI does not explicitly use a human visual system model, it performs significantly better than the widely used distortion metric mean squared error for various types of image distortion. A feasibility is to keep the convergence of SART and ASD-POCS reconstruction for 5 iterations and MLEM reconstruction for 2 iterations (Figure 4).

The amplification of noise is a characteristic of nonregularized algorithms, such as MLEM. As high-frequency noise in the data is amplified by each iteration of the MLEM algorithm, few iterations may be optimum for the detection of low-contrast objects, such as small masses [3]. In the SART algorithm, the linear attenuation coefficient of each voxel is simultaneously updated using all rays in a single projection (regularized algorithm). The number of imaged volume updates in a single iteration is equal to the number of projections [23]. Considering these factors, we believe that the difference between RMSE and QI is associated with the difference between SART and MLEM algorithms.

### 2.7. Case Evaluation

In this study, AEC exposure was used to compare different DBT reconstruction methods in a clinical case evaluation. The cases were evaluated using structural similarity (SSIM) [29], where local patterns of luminance- and contrast-normalized pixel intensity were compared to determine the SSIM index of contrast preservation. This image quality metric is based on the assumed suitability of the human visual system for extracting structure-based information.

The SSIM index between pixel values *x* and *y* was calculated as follows:

(2) $$\begin{array}{c} SSIM\left(\;x,y\;\right)={\left[\;l\left(\;x,y\;\right)\;\right]}^{\alpha }\cdot {\left[\;c\left(\;x,y\;\right)\;\right]}^{\beta }\cdot {\left[\;s\left(\;x,y\;\right)\;\right]}^{\gamma } \end{array}$$

where *l* is the luminance, *c* is the contrast, and *s* is the structure. Subsequently,

(3) $$\begin{array}{c} \alpha =\beta =\gamma =1.0. \end{array}$$

The mean SSIM (MSSIM) was then used to evaluate the overall image quality:

(4) $$\begin{array}{c} MSSIM\left(\;X,Y\;\right)=\frac{1}{M}\overset{M}{\underset{j=1}{\sum }}SSIM\left(\;x_{i},y_{j}\;\right) \end{array}$$

where *X* and *Y* are the reference [reconstructed image (in-focus plane) from 15 projections] and objective [reconstructed image (in-focus plane) from seven projections] images, respectively; *x*_*i*_ and *y*_*j*_ are the image contents at the *j*th pixel; and *M* is the number of pixels in the image.

Details of each case are listed below.

*Case 1*. In a 56-year-old woman with diagnosed ductal carcinoma in situ, the following imaging parameters were used: voltage, 30 kV; tube current, 61; thickness, 46 mm; AGD, 1.75 (15 projections). 

*Case 2*. In a 62-year-old woman with a diagnosis of scirrhous, the following parameters were used: voltage, 29 kV; tube current, 47; thickness, 39 mm; AGD, 1.32 (15 projections). 

*Case 3*. In an 81-year-old woman with a diagnosis of solid tubular carcinoma, the following parameters were set: voltage, 29 kV; tube current, 48; thickness, 41 mm; AGD 1.29 (15 projections).

## 3. Results

**Figure 5:**
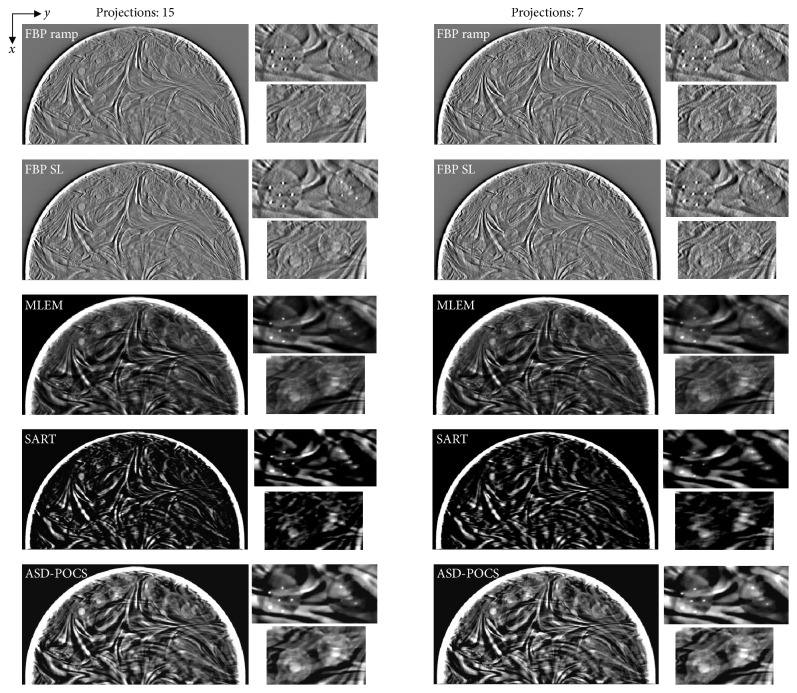
Comparisons between different projection number (normal: 15, half: seven) images obtained using each tomosynthesis reconstruction algorithm in the in-focus plane. The X-ray source was moved horizontally along the image. Zoomed images: microcalcifications, spheroidal masses. For corresponding images, the IR (MLEM, SART, and ASD-POCS) images are displayed at the same window width and level, whereas the FBP images have a larger window widths because the backgrounds are less flattened and the gray levels in larger areas would be out of scale in narrower windows.

Figure 5 presents images of the BR3D Phantom obtained using each reconstruction algorithm. Compared with the FBP algorithm, the IR algorithms tended to yield slightly higher noise levels as the projection number decreased, although image quality deterioration was not observed. The FBP algorithm exhibited good MC detection ability but also generated remarkable false images from the peripheries of the MCs.

**Figure 6:**
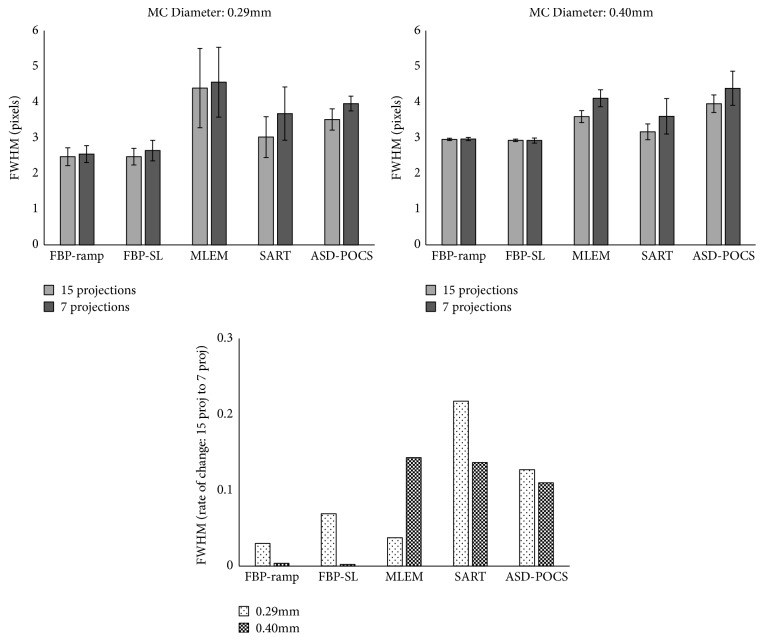
Comparisons of the full width at half-maximum (FWHM) and rate of change between normal (15) and half (seven) projections in the in-focus plane images obtained via tomosynthesis under different projection numbers and generated using different reconstruction algorithms. The error bar represents the standard error.

We also compared the FWHM of each reconstructed image obtained using different projection numbers for the in-focus plane (Figure 6). Here, the FBP algorithm yielded the best spatial resolution, whereas this parameter tended to deteriorate while using the ASD-POCS algorithm to reconstruct a reduced number of projections. Furthermore, the number of projections but not the MC size affected the FWHM rate of change.

**Figure 7:**
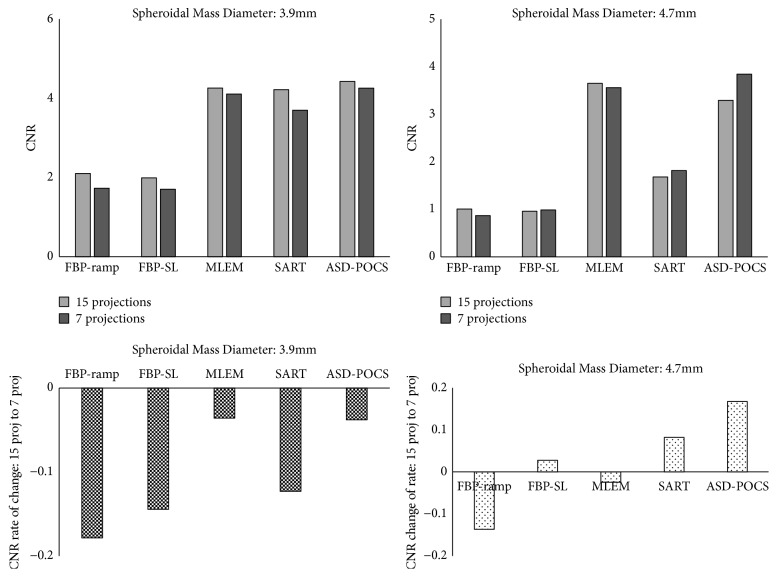
Comparisons of the contrast-to-noise ratio (CNR) and rate of change between the normal (15) and half (seven) projections in the in-focus plane images obtained via tomosynthesis under different projection numbers and generated using different tomosynthesis reconstruction algorithms.

We further compared the CNR of each reconstructed image obtained using different projection numbers for the in-focus plane (Figure 7). Notably, the ASD-POCS algorithm yielded high-contrast images, regardless of the simulated mass lesion size and projection number. With the FBP algorithm, the contrast degradation increased at reduced projection numbers when generating images of 3.9-mm spheroidal masses. With the MLEM and ASD-POCS algorithms, contrast deterioration associated with a decreased projection number had only a small effect. For 4.7-mm spheroidal masses, the CNR tended to increase as the projection number decreased when using the FBP(SL), SART, and ASD-POCS algorithms and tended to decrease when using the FBP (ramp) and MLEM algorithms.

**Figure 8:**
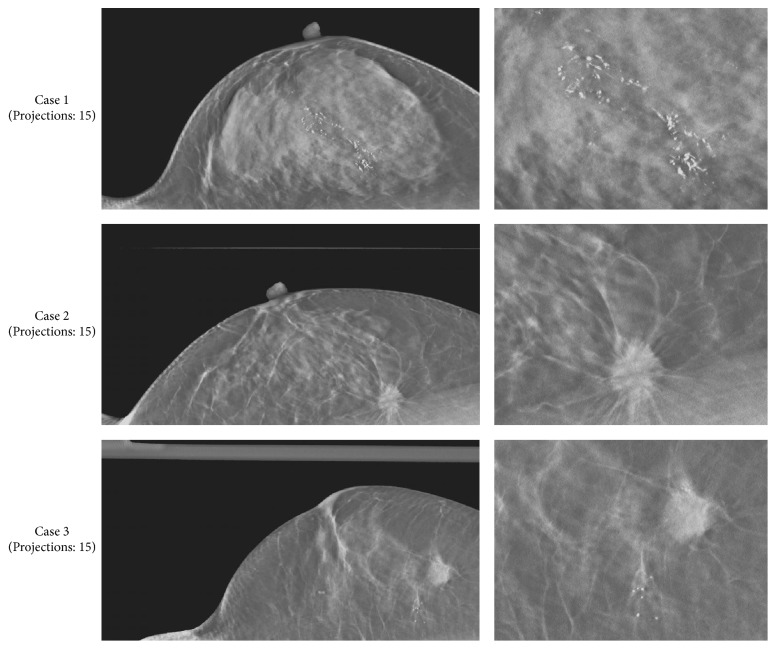
Comparisons among clinical case images (in-focus plane) obtained via actual filtered back projection reconstructions from a digital breast tomosynthesis scanner (Selenia Dimensions, 15 projections). The scanner FBP images for the corresponding image are displayed with the same window width and window level (top: case 1, middle: case 2, and bottom: case 3).

**Figure 9:**
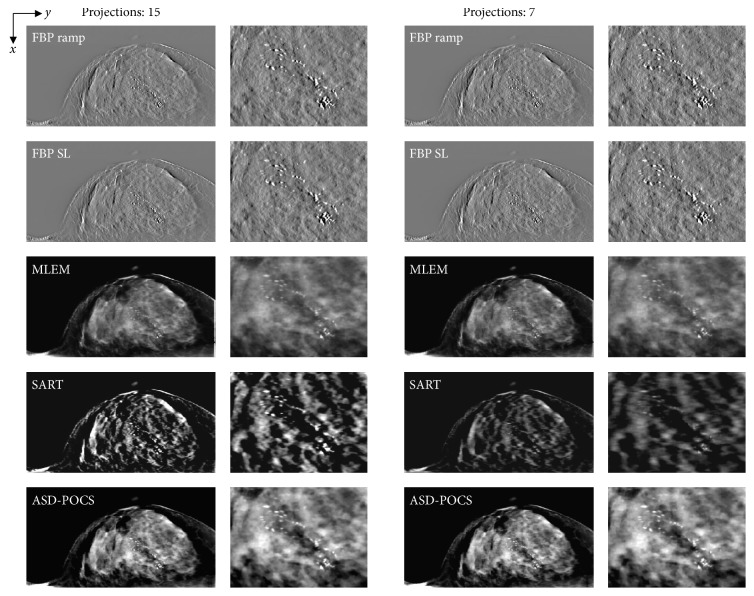
Case 1. Comparisons between images obtained at different projection numbers (normal: 15, half: seven) using each tomosynthesis reconstruction algorithm in the in-focus plane. The X-ray source was moved horizontally along the image. Zoomed images depict the lesion areas. For each corresponding set, IR (MLEM, SART, and ASD-POCS) images are displayed at the same window width and level, whereas the FBP images have a larger window widths because the backgrounds are less flattened and the gray levels in larger areas would be out of scale in narrower windows.

**Figure 10:**
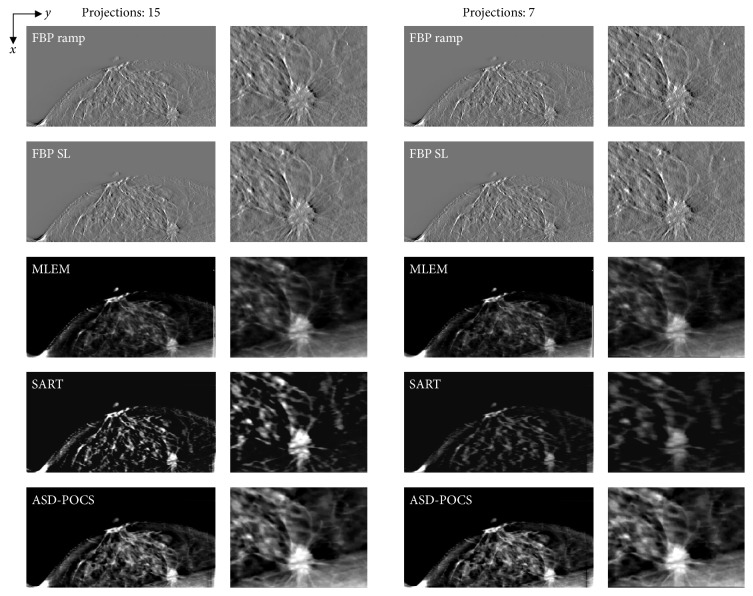
Case 2. Comparisons between images obtained at different projection numbers (normal: 15, half: seven) using each tomosynthesis reconstruction algorithm in the in-focus plane. The X-ray source was moved horizontally along the image. Zoomed images depict the lesion areas. For each corresponding set, IR (MLEM, SART, and ASD-POCS) images are displayed at the same window width and level, whereas the FBP images have a larger window widths because the backgrounds are less flattened and the gray levels in larger areas would be out of scale in narrower windows.

**Figure 11:**
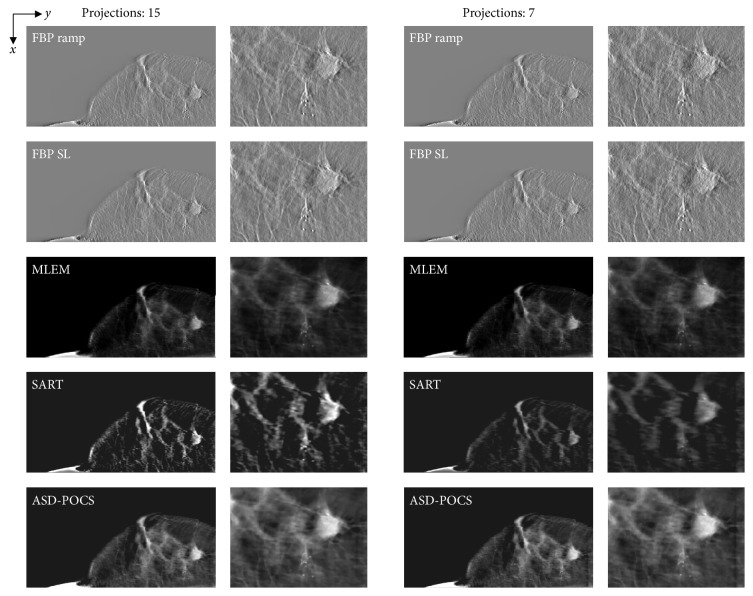
Case 3. Comparisons between images obtained at different projection numbers (normal: 15, half: seven) using each tomosynthesis reconstruction algorithm in the in-focus plane. The X-ray source was moved horizontally along the image. Zoomed images depict the lesion areas. For each corresponding set, IR (MLEM, SART, and ASD-POCS) images are displayed at the same window width and level, whereas the FBP images have a larger window widths because the backgrounds are less flattened and the gray levels in larger areas would be out of scale in narrower windows.

**Figure 12:**
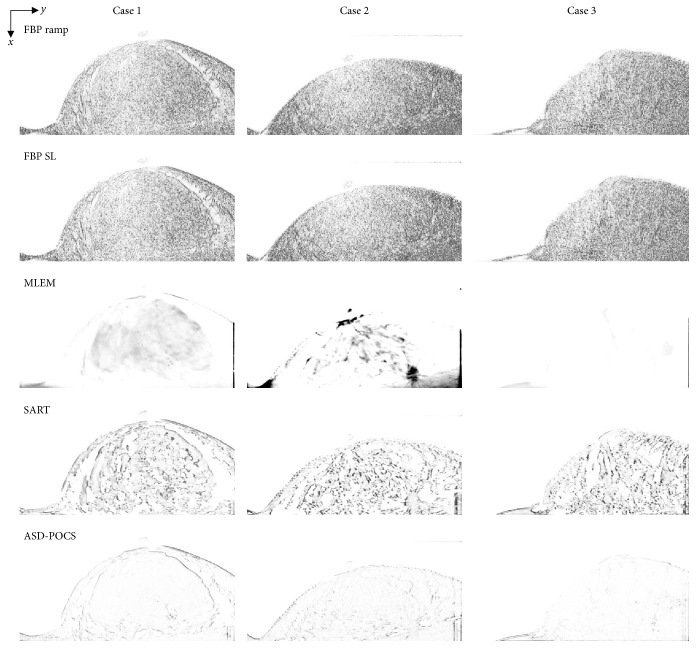
Comparisons of structural similarity among images obtained from each case and using each tomosynthesis reconstruction algorithm in the in-focus plane. The X-ray source was moved horizontally along the image. Images are in grayscale; white and black indicate high and low structural similarity, respectively. The window width and level in each display are varied to allow a visual comparison of the contrast and background gray level. Corresponding IR (MLEM, SART, and ASD-POCS) images are displayed at the same window width and level.

**Figure 13:**
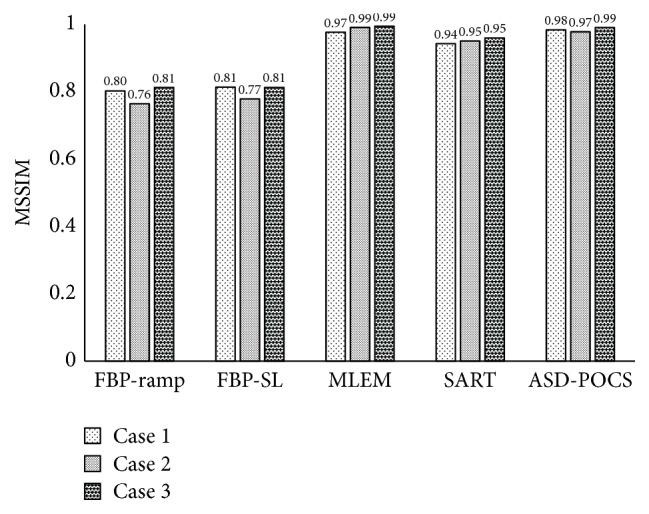
Comparisons of mean structural similarity among in-focus plane images obtained via tomosynthesis data from clinical cases under normal (15) and half projections (seven) and using each tomosynthesis reconstruction algorithm.

Reconstructed images of three clinical cases, which were obtained using actual FBP reconstructed images (in-focus plane) from the scanner (Selenia Dimensions), are presented as reference data in Figure 8. Reconstructed clinical case images obtained using each reconstruction algorithm are presented in Figures 9–11, while Figure 12 compares the SSIM images obtained with each reconstruction algorithm using either 15 or seven projections for the in-focus plane.  Figure 13 compares the MSSIM of each reconstructed clinical case image obtained in the in-focus plane. Images reconstructed using the ASD-POCS and MLEM algorithms and a reduced number of projections were highly structurally similar, suggesting that the former could potentially be used in dose reduction initiatives while preserving contrast.

## 4. Discussion

Our empirical results obtained using various reconstruction algorithms demonstrate that the ASD-POCS algorithm can preserve image contrast even when using reduced projection data. Accordingly, the ASD-POCS algorithm could potentially be used to reduce radiation doses to patients.

The outermost repeat-until loop instruction of the ASD-POCS algorithm contains two main components: an adjustment toward data consistency via the POCS step loop and the steepest descent toward lower-TV images. The algorithm is effective when each POCS step involves multiple small descent substeps, particularly during the early iterations [20]. TV-minimization assumes that the true image is piecewise and relatively uniform, whereas noise and artifacts appear as fluctuations, or peaks and valleys; accordingly, noise and artifact-corrupted images would have relatively larger TV values because TV is defined as the sum of first-order derivative magnitudes [22]. In contrast, compared with the SART algorithm MSSIM results similar to the ASD-POCS algorithm have shown preserved contrast, but a large change rate from the FWHM and low-contrast detection compared with the ASD-POCS algorithm. We suggest that these results are attributable to the inability of the SART algorithm to correct increases in noise. Moreover, incorrect assumptions in the reconstruction models lead to the active introduction of artifacts, whereas TV suppresses the high-frequency components of artifacts introduced by data consistency.

In general, image artifacts are caused by a loss of the largest normal contributions from artifact-free voxels. As these voxels normally produce original contributions, their values decrease slightly after the largest normal contribution has been omitted. Accordingly, a single abnormal contribution within a voxel is resolved while all other contributions are retained, including the largest normal contribution; voxels containing such abnormalities therefore tend to exhibit higher values than their neighboring artifact-free voxels, leading to the appearance of objects in which artifact-free voxels are more noticeable against the background. This phenomenon is a drawback of the FBP algorithm, and consequent artifacts are conspicuous when compared with artifact-free images.

DT image quality depends on several factors, including size, shape, density, atomic number, and the size and shape of the object cross-section. Highly attenuating objects yield streak artifacts (dark-band artifacts) on DBT acquisitions, which adversely affect image quality. Additionally, beam hardening and scattering have significant effects, particularly on highly attenuating objects from MCs. Accordingly, noise-induced streak artifacts primarily affect image quality. In such cases, the IR reconstruction algorithm, which is thought to adequately address quantum noise [30], appears to be a promising approach to the reduction of artifacts stemming from MCs with relatively high atomic numbers.

SART does not imply an even distribution of noise across an image. Rather, SART uses an algebraic matrix to selectively identify and subtract noise from an image according to a mathematical model. However, each iteration of the reconstruction algorithm amplifies high-frequency noise within the data. Therefore, the MLEM algorithm may be optimal for detecting low-contrast objects [3].

We further found that images generated using the ASD-POCS algorithm with a reduced projection number exhibited deteriorated spatial resolution. TV-based approaches uniformly penalize the image gradient, regardless of the image structure. Therefore, oversmoothing of the reconstructed image remains a major concern, despite the advantages of using a TV norm as the regularization term [31]. Frequent oversmoothing of the edges of the reconstructed image causes the loss of low-contrast information [32].

Most studies have evaluated breast imaging at different radiation doses [33]. We believe that investigations of the relationship between normal and half projections and contrast preservation are useful for determining the feasibility of radiation dose reduction and hope that our study results serve as a guideline for image reconstruction under reduced projection data conditions. However, our study had several limitations. First, we did not test actual mammary gland tissues. However, we believe that the BR3D Phantom is an accurate representation of actual mammary gland tissues. Second, we did not perform an observational study. In future, we plan to conduct such a study to investigate the correlations among physical evaluation parameters (e.g., spatial resolution and contrast). Third, the phantom thickness was fixed at 4 cm. In future evaluations, other phantom thicknesses will be needed to confirm the utility of the algorithm. Despite these limitations, we believe that our results can serve as reference data and thus assist physicians with contrast preservation while reducing radiation exposure.

## 5. Conclusion

This study evaluated the ASD-POCS algorithm as a novel technique for contrast preservation in DBT images obtained under a reduced projection number. Our findings suggest that the ASD-POCS algorithm could be used to reconstruct dose-reduced images. As this approach exploits* a priori* knowledge about contrast preservation and noise reduction, we presume that the ASD-POCS algorithm will enhance the clinical application of DBT in medical imaging, wherein these parameters are a major focus of interest.

## Data Availability

No data were used to support this study.
